# Allergies: The New Lore of Spores

**DOI:** 10.1289/ehp.114-a576

**Published:** 2006-10

**Authors:** Julie Wakefield

When it comes to allergies, not all fungi are created equal, according to a study by University of Cincinnati researchers published in the September 2006 issue of *Pediatric Allergy and Immunology*. Exposure to certain fungal spores can make children more susceptible to developing allergies to mold, pollen, dust mites, pet dander, or foods, the study revealed. On the other hand, exposure to other types of fungal spores may be protective.

Fungal samples were collected in 2003 and 2004 from the homes of 144 infants under age 10 months as part of the five-year Cincinnati Childhood Allergy and Air Pollution Study, supported by the NIEHS. Fungal measurement included long-term air sampling—48 hours, compared to the 5–10 minutes typical of such studies—which improved exposure assessment. The team then analyzed the spore samples, comparing the breakout with allergy symptoms exhibited by the infants (such as sneezing and runny nose) and skin-prick tests for 17 allergens with specific fungal spore counts.

Children who were exposed to higher levels of spores from *Basidiomycota* (club fungi) and *Penicillium/Aspergillus* (whose spores are very similar) were more likely to develop multiple allergies, says coauthor Tiina Reponen, a professor in the University of Cincinnati Department of Environmental Health. Those exposed to *Basidiomycota* were more likely to exhibit allergy symptoms; those exposed to *Penicillium/Aspergillus* and *Alternaria* (one of the most common fungi in outdoor air) were more likely to have a positive skin-prick test.

Meanwhile, exposure to *Cladosporium* (a black mold) had the opposite association, with exposed children testing positive for sensitivity to fewer allergens. This contrasts with the experience of adults, in whom *Cladosporium* has been associated with greater allergic sensitization.

The researchers did not find any correlation between the total fungi count and allergies. “The [observed] associations would have been missed if the exposure was assessed by using the total [fungal spore] count only,” Reponen says. The study indicates that the relationship between exposure to airborne fungal spores and health effects is more complicated than researchers believed.

The indoor environment is indeed complicated. According to the authors, allergens can mix with pollutants and toxicants in synergistic ways, effects that were not addressed in this study. “We believe that contrasting relationships among the various fungal genera to the health outcomes investigated in this study might actually cancel the effect that *total* concentration may have on these outcomes,” the researchers write.

Although researchers still know little about how infants develop allergies to environmental agents, the new study offers new insights into the health effects of fungi, according to Zalman Agus, associate dean of continuing medical education at the University of Pennsylvania. The study lends credence to the so-called hygiene hypothesis, which posits that an ultraclean environment may wipe out innocuous organisms and collaterally alter some parts of children’s developing immune systems.

Yet researchers don’t know how some microbes might induce a protective effect. One hypothesis is that *Cladosporium* exposure may inhibit the response of Th2 lymphocytes, white blood cells that block dangerous microbes or foreign organisms from invading the body’s cells.

Longer-term follow-up of this cohort will better elucidate the clinical implication of the findings, Reponen says. In the meantime, the researchers advise in the paper that “clinicians and researchers should be attentive to the composition of the fungal spore profile and the respective concentrations of the fungal genera present rather than the total or culturable spore count alone.”

## Figures and Tables

**Figure:**
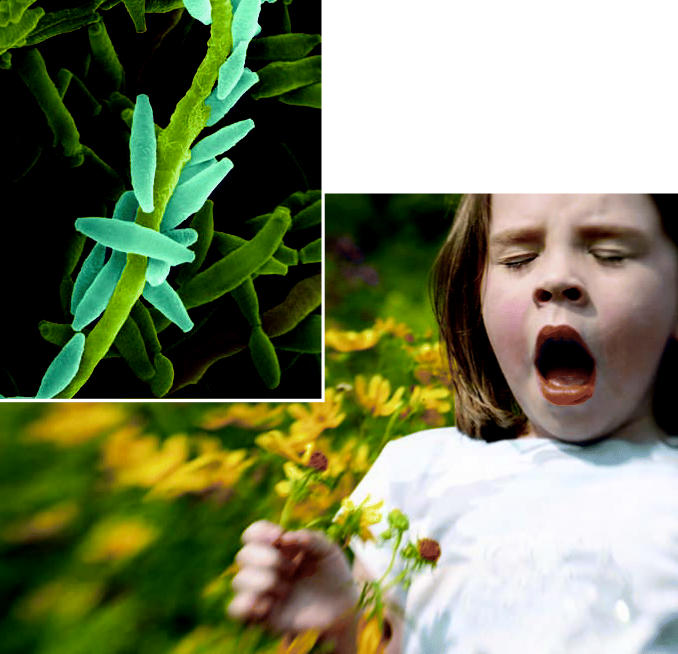
Fungi and future effects Exposure to spores of certain fungi may predispose children to developing more allergies, while exposure to others, such as those of the mold *Cladosporium* (inset), may confer a protective effect, resulting in sensitivity to fewer allergens.

